# Role of Recent Therapeutic Applications and the Infection Strategies of Shiga Toxin-Producing *Escherichia coli*

**DOI:** 10.3389/fcimb.2021.614963

**Published:** 2021-06-29

**Authors:** Su-bin Hwang, Ramachandran Chelliah, Ji Eun Kang, Momna Rubab, Eric Banan-MwineDaliri, Fazle Elahi, Deog-Hwan Oh

**Affiliations:** ^1^ Department of Food Science and Biotechnology, College of Agriculture and Life Sciences, Kangwon National University, Chuncheon, South Korea; ^2^ School of Food and Agricultural Sciences, University of Management and Technology, Lahore, Pakistan

**Keywords:** Shiga toxin-producing *Escherichia coli* (STEC), Shiga toxin, infection, symbiotic, antimicrobial agents, therapies

## Abstract

Shiga toxin-producing *Escherichia coli* (STEC) is a global foodborne bacterial pathogen that is often accountable for colon disorder or distress. STEC commonly induces severe diarrhea in hosts but can cause critical illnesses due to the Shiga toxin virulence factors. To date, there have been a significant number of STEC serotypes have been evolved. STECs vary from nausea and hemorrhoid (HC) to possible lethal hemolytic-based uremic syndrome (HUS), thrombotic thrombocytopenic purpura (TTP). Inflammation-based STEC is usually a foodborne illness with Shiga toxins (*Stx 1* and *2*) thought to be pathogenesis. The STEC’s pathogenicity depends significantly on developing one or more Shiga toxins, which can constrain host cell protein synthesis leading to cytotoxicity. In managing STEC infections, antimicrobial agents are generally avoided, as bacterial damage and discharge of accumulated toxins are thought the body. It has also been documented that certain antibiotics improve toxin production and the development of these species. Many different groups have attempted various therapies, including toxin-focused antibodies, toxin-based polymers, synbiotic agents, and secondary metabolites remedies. Besides, in recent years, antibiotics’ efficacy in treating STEC infections has been reassessed with some encouraging methods. Nevertheless, the primary role of synbiotic effectiveness (probiotic and prebiotic) against pathogenic STEC and other enteropathogens is less recognized. Additional studies are required to understand the mechanisms of action of probiotic bacteria and yeast against STEC infection. Because of the consensus contraindication of antimicrobials for these bacterial pathogens, the examination was focused on alternative remedy strategies for STEC infections. The rise of novel STEC serotypes and approaches employed in its treatment are highlighted.

## Introduction

### Shiga Toxin-Producing *Escherichia coli* (STEC) Gastroenteritis and Hemolytic Uremic Syndrome

**Figure 1:**
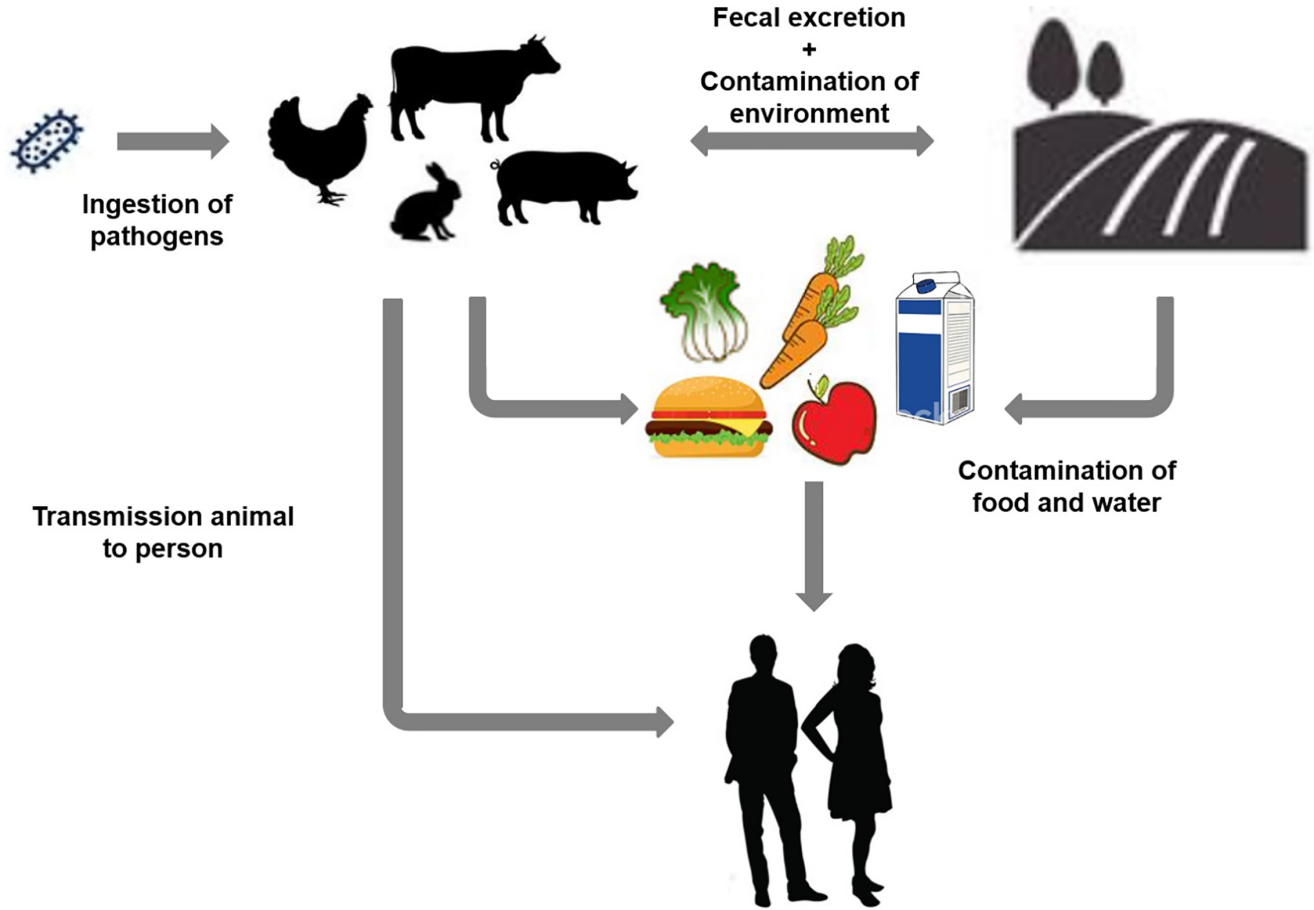
Schematic representation of different contamination sources and transmission of Shiga toxin-producing *Escherichia coli* infection based on various environmental factors.

Enteropathogens induce numerous diseases, most of them featuring colon distress symptoms. Enteropathogenic bacteria such as diarrhea-causing *Escherichia coli* (*E. coli*) and species of the genera *Salmonella, Shigella*, *Klebsiella*, and *Yersinia* are responsible for different types of gastrointestinal disorders. STEC is a prominent bacterial pathogen reported globally (Bhunia, 2018). Some *E. coli* naturally reside in animals and humans’ colonic tract and are considered beneficial gut bacteria. However, most of the pathogenic strains of *E. coli* such as STEC are responsible for several colon infections (Bron et al., 2017). STEC is one of the six major classifications (pathotypes) of diarrheagenic *E. coli*. This differentiation depends on medical syndromes, symptoms, epidemiology, the presence of antigen type Stx1 and Stx2 virulence factors, and interaction with epithelial cells (Hwang et al., 2018). Infection with most types of enteropathogenic *E. coli* causes watery diarrhea (**Figure 1**). Many enteric infections lead to a short-lived dysfunction of the gastrointestinal system. In extreme cases, a severe disorder can occur based on specific pathogenic infections such as that with STEC (Bron et al., 2017; Hwang et al., 2018).

STEC serves as a source of food and water-borne outbreaks that contribute to life-threatening infections. STEC infection outcomes can range from mild to significant symptoms of hemorrhagic colitis (HC) and hemolytic-based uremic syndrome (HUS). Certain STEC strains are also designated as enterohemorrhagic *E. coli* (EHEC) due to their human virulence factors (Rivas et al., 2016). EHEC strains belong to the STEC subtype and are distinguished by specific serotypes, often correlated with epidemic and severe clinical disease (Mora et al., 2012). EHEC-based O157:H7 strain was reported in the center for disease control and prevention (CDC) study on microbiological findings of raw ground beef products, which was linked to several cases of HC and HUS (Fedio et al., 2011). As a result, public health and regulatory responses were primarily based on this serogroup. Cumulative evidence from various countries has shown in recent years that up to 40 to 70 percent of human EHEC diseases are induced by non-O157 EHEC (Delannoy et al., 2013).

## Emerging STEC Serotypes

Recent research has shown that the number of STEC infections other than O157 often exceeds the number of STEC O157 infections (Gould et al., 2009; Gould et al., 2013). The HUS-associated STEC list and their non-motile derivatives were therefore expanded in **Table 1A**. These are the seven STEC priority serotypes most often associated with HC and HUS infections and sporadic cases worldwide (Stanford et al., 2018); USDA study on the classification of non-O157 STEC from meat products are reported in **Table 1B**.

**Table 1A:** A description of the global reports of outbreaks of two cases or more of non-O157 strains of Shiga toxin-producing *E. coli* along with the reported frequency of dysentery and hemolytic uremic syndrome where these data were available, and the implicated vehicle of transmission, 1995ֲ017. (Copyright obtained from Valilis et al., 2018).

Years	Number of confirmed cases	Serogroups/types	Median number of people per outbreak (range)	Number reporting dysentery (%)	Implicated vehicle of transmission	Number reporting HUS (%)
**1995–1999**	**183**	**O26:H11, O111:(H-,H8), O118:H2**	**25/(2–131)**	**6/57 (11%)**	**Ice in open barrels, serving utensil, dry fermented sausage**	**16/183 (8.7%)**
1990	5	O111	1/5	1/5	Private home/Family cluster	
1994	18	O104	0/18	0/18	Pasteurized Milk	
1998	8	O121	unknown	unknown	Camp	
1999	55	O111	2/55	2/55	Salad Bar, Ice from barrel	
1999	11	O121	3/11	3/11	Lake Water	
1999	2	O121	0/1	0/1	Daycare	
**2000–2004**	**26**	**O26:H11, O148:H8**	**11/(2–13)**	**0/26 (0%)**	**Mutton, beef**	**2/26 (7.7%)**
2000	61	O111	0/59	0/59	Animal contact (calves)	
2000	18	O145	2/18	2/18	Water-based punch	
2001	4	O26	0/4	0/4	Lake Water	
2001	31	O111,O-rough	0/25	0/25	Animal Contact (Calves)	
2001	3	O111	3/3	3/3	Family cluster (animal exposure reported for one patient)	
2001	3	O111	0/3	0/3	Daycare	
2004	213	O111	0/212	0/212	Unpasteurized Apple Cider	
**2005–2009**	**221**	**O26:(H11), O45, O103:H25, O104:H4, O111, O145:H28**	**16/(3–156)**	**93/137 (68%)**	**Ice cream, farm animals, eating outside of home, restaurant, beef sausage, mutton**	**34/91 (37%)**
2005	52	O45	0/52	0/52	Ill Food Worker(s)	
2005	4	O26	unknown	unknown	Daycare	
2006	42	O121	3/42	3/42	Lettuce	
2006	5	O26	0/4	0/4	Berries	
2006	5	0121	4/5	4/5	Daycare	
2006	11	O45	0/11	0/11	Animal contact (goats)	
2006	3	O165	0/3	0/3	Correctional facility	
2007	23	O111	0/23	0/23	Private home (ground beef)	
2007	8	O111	0/8	0/8	Daycare	
2010–2014	184	O26:(H11), O103:H2, O104:H4, O111:H8, O121, O145:(NM)	25/(2–35)	20/184 (11%)	Raw clover sprouts, Farm Rich brand frozen products, dairy products, cattle, person-to-person, venison, romaine lettuce	26/184 (14%)
	3816	O104:H4a	3816	141/161 (88%)	Sprouts	845/3816 (22%)
2015–2017	60	O26	30/(5–55)	0/60 (0%)	Multiple restaurant chains	0/60 (0%)

*Excludes any isolates for which serogroup could not be determined (including isolates in unknown, undetermined, and rough categories). HUS, hemolytic uremic syndrome; STEC, Shiga toxin-producing E. coli.

**Table 1B:** Non-O157 STEC isolates characterized at the National *Escherichia coli* Reference Laboratory, by serogroups.

Serogroup			Number of isolates reported, 1995-2020			Percentage of total isolates serogroup	
14			7			0.2%	
22			7			0.2%	
88			7			0.2%	
91			60			1.5%	
76			52			1.3%	
165			45			1.1%	
228			28			0.7%	
174			27			0.7%	
123			23			0.6%	
177			22			0.6%	
153			21			0.5%	
28			20			0.5%	
178			10			0.3%	
63			9			0.2%	
7			8			0.2%	
2			7			0.2%	
**26**			**918**			**23.2%**	
**103**			**806**			**20.4%**	
**111**			**643**			**16.3%**	
45			290			7.3%	
121			248			6.3%	
145			179			4.5%	
69			71			1.8%	
118			71			1.8%	
117			6			0.2%	
175			6			0.2%	
84			19			0.5%	
128			19			0.5%	
146			18			0.5%	
113			17			0.4%	
119			15			0.4%	
8			14			0.4%	
55			14			0.4%	
172			12			0.3%	
130			10			0.3%	
156			10			0.3%	
126			7			0.2%	
9			6			0.2%	
110			6			0.2%	
112			6			0.2%	
179			6			0.2%	
6			5			0.1%	
43			5			0.1%	
71			5			0.1%	
141			5			0.1%	
181			5			0.1%	
1			4			0.1%	
33			4			0.1%	
50			4			0.1%	
80			4			0.1%	
98			4			0.1%	
116			4			0.1%	
132			4			0.1%	
166			4			0.1%	
51			3			0.1%	
60			3			0.1%	
73			3			0.1%	
79			3			0.1%	
82			3			0.1%	
86			3			0.1%	
109			3			0.1%	
125			3			0.1%	
162			3			0.1%	
163			3			0.1%	
168			3			0.1%	
5			2			0.1%	
11			2			0.1%	
18			2			0.1%	
20			2			0.1%	
21			2			0.1%	
25			2			0.1%	
38			2			0.1%	
42			2			0.1%	
49			2			0.1%	
53			1			0.0%	
61			1			0.0%	
70			1			0.0%	
87			1			0.0%	
96			1			0.0%	
101			1			0.0%	
105			1			0.0%	
115			1			0.0%	
131			1			0.0%	
74			2			0.1%	
75			2			0.1%	
77			2			0.1%	
85			2			0.1%	
100			2			0.1%	
104			2			0.1%	
124			2			0.1%	
136			2			0.1%	
137			2			0.1%	
143			2			0.1%	
149			2			0.1%	
158			2			0.1%	
160			2			0.1%	
3			1			0.0%	
4			1			0.0%	
12			1			0.0%	
19			1			0.0%	
24			1			0.0%	
27			1			0.0%	
52			1			0.0%	
134			1			0.0%	
135			1			0.0%	
140			1			0.0%	
150			1			0.0%	
151			1			0.0%	
152			1			0.0%	
154			1			0.0%	
180			1			0.0%	

*Data represented from CDC Bacterial Foodborne and Diarrheal Disease National Case Surveillance Annual Reports, 2003-2020.

## Mode of Transmission of STEC and the Effect of Shiga Toxin in Humans and Animals

STEC leads to fatal inflammation in the host as a sign of Shiga-based toxins’ expression. STEC, comprising strains of the serogroups (**Table 1**), causes severe diarrhea, hemorrhagic colitis (HC), and can also lead to life-threatening diseases like hemolytic uremic syndrome (HUS) (Singh et al., 2015) (**Table 1**). The frequency of non-O157 STEC-based cases in the United States (U.S) was unclear; based on the symptoms, the researchers have quantified the level of infection in the human feces (Newell and La Ragione, 2018). Mostly, non-STEC strains (specifically O26,O45,O103,O111, and O145 serogroups) showed similar virulence and biochemical characteristic with the O157 strain as per US research reports (Gould et al., 2013). In 2020 as per the Centers for Disease Control and Prevention (CDC) FoodNet Data and Reports indicated that the level of non –STEC O157 was found to be significantly higher than the STEC *E. coli* (Joseph et al., 2020). Besides, the food Net report states that among 451 non-O157 STEC reported cases 80 percent was children, and the rest 20% was adult (Joseph et al., 2020). From 2000-2010, FoodNet reported 1,842 instances of non-STEC O157 infection were (**Table 1A**).

Based on the epidermic of non-O157 STEC reported by outbreak Surveillance System (FDOSS) which identified over 1,500 illnesses confirmed cases of non-O157 STEC outbreaks on November 4, 2020 (Thierry et al., 2020). Most of the epidemic was caused by non 0157 serotype strains but correlated with other enteropathogens (EFSA BIOHAZ Panel et al., 2020) . The most frequent outbreak of non 0157 STEC serotype reported among 120 serogroups was determined as follows O26, O111, and O121 (**Table 1B**) (Blankenship et al., 2020). The pathogenicity with *Shigella species* and STEC *E. coli* was almost similar but they are varied in the symptoms, metabolic traits, and severity of illness (Bommarius et al., 2013; Chou et al., 2013; Merkx-Jacques et al., 2013; Michelacci et al., 2017; Smati et al., 2017; Yun et al., 2017). The entero-aggregative STEC outbreak reported with 790 cases of HUS and 3128 non- HUS cases in Germany (May 2011) indicated a lethal HUS percentage (Cheung et al., 2011). Noval STEC strains (new serotypes) were reported for HUS cases (Bae et al., 2006). Among the previous outbreak, reports consisted that the major non-O157 STEC such as O26, O45, O103, O111, O121, and O145, likewise among the STEC the most frequent reports in 2020 such as O26:H11, O111:H8, and O121:H19 serogroups (Taylor et al., 2013) (**Supplementary Table 1**).

The pathogenic mechanisms of STEC merited further investigation. As per the proven theory, it was predicted that *Shigella* strains were evolved and it forms the ancestral for infectious virulent *E. coli* (Welch et al., 2002; Paauw et al., 2015; Njamkepo et al., 2016; Dunne et al., 2017). In contrast, Enteroinvasive *E. coli* (EIEC) are thought to have evolved later than *Shigella* and from widely diverged strains of *E. coli* (Lan et al., 2004). Additional research is required to characterize the virulent effects of STEC, and it is hoped this could prevent the evolution of novel strains that are more virulent or difficult to treat and could pose a serious human health threat. Several animal models have been proposed for studying EHEC infection (Panda et al., 2010; Golan et al., 2011; Mohawk and O’Brien, 2011). Some of the infectious mechanism and virulence factors are yet to be determined for the EHEC due to the lack of an efficient animal model system which hampers the pathway mechanism (Franz et al., 2014) (**Supplementary Table 2**).

## STEC Attachment and Pathogenicity in the Intestinal Environment

**Figure 2:**
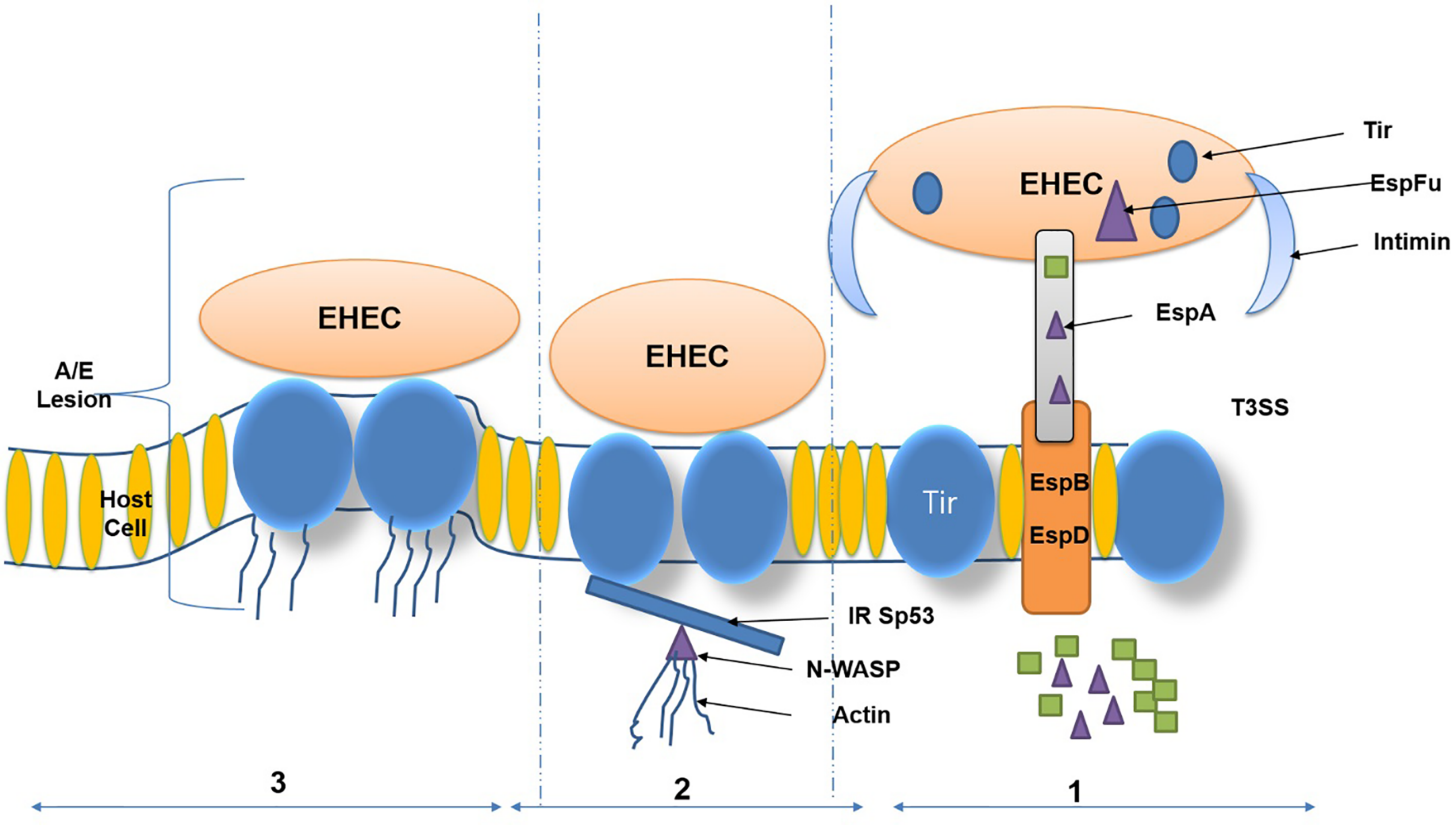
Virulence factors and attaching and effacing (A/E) lesions of Enterohemorrhagic *Escherichia coli* (EHEC). (Copyright obtained from Saeedi et al., 2017).

Diverse adhesive assemblies connected to *E. coli* O157:H7 cells influence the bacteria’s adhesion to intestinal epithelial cells. These morphological attachment-based structures include fimbria, which is responsible for binding and multiplication. These adhesions of STEC bacteria facilitate the surface attachment of bacteria to human intestinal epithelial cells, which possess glycoprotein as an associate protein (Suzaki et al., 2002; Uchida, 2003). The adhesion mechanism is directly linked to virulence factors of *E. coli* OH157:H7 and leads to inflammation (lesions in colon inner wall). EHEC harbors a Type 3 secretion system (*T3SS*) and its secreted proteins, including *EspD, EspB, EscF,* and *EspA*. The *T3SS* and its secreted proteins are encoded on the locus of enterocyte effacement (LEE) pathogenicity. The over-expression was regulated by *LEE*-encoded regulator (Ler) upregulates LEE-encoded virulence genes. Some of the effector proteins imitate host ligands and receptors involved in attachment with epithelial cells. The primary function of the translocated proteins, which trigger activation of neural syndrome protein (N-WASP) such as the translocated intimin receptor (Tir), and an adaptor-like protein *EspFU* (Gao et al., 2009). The solid human immune response generated against intimin receptor*, EspA*, and *EspB* has led to these bacterial proteins being considered potential vaccine candidates (**Figure 2**).

## Correlation of Antimicrobial Resistance With Increased Toxin Gene Expression in STEC

Previous reports have described resistance mechanism in *E. coli* O157:H7 and other STEC strains mainly evolved from animal reservoir-based environmental sources (**Supplementary Table 4**). The principal reason for increasing reports on antibiotic resistance is the overuse of antibiotics in agricultural-based regions, which leads to the development of multidrug resistance in bacteria. In STEC, the *Stx* gene is responsible for the production of Shiga toxin (**Supplementary Table 5**). Chloramphenicol was commonly used to suppress the growth of STEC, but along with other drugs such as sulfonamides, quinolones, and fluoroquinolones leads to enhanced toxin production as a result of over-expression of the *Stx* gene (Schroeder et al., 2002; Amézquita-López et al., 2016). Besides, STEC was commonly found to be resistant to certain antibiotics such as tetracyclines and aminoglycosides (Uemura et al., 2003; Bai et al., 2016) (**Supplementary Table 6**).

The horizontal gene transfer of resistance is a multidrug resistance (MDR) dissemination mechanism since virulence and antibacterial factors-based genes can be found in clusters and transmitted together to the recipient (Bello-López et al., 2019). Various theories of recombination, transition, or intracellular transduction may transfer these genes. Under environmental circumstances, the most effective transfer mechanisms in bacteria appear to be conjugation and transduction (Muniesa et al., 2013). Such tools often involve integrons, which are mobile genetic elements that acquire gene cassettes for antimicrobial resistance. Integrons are described as genetic units that include site-specific recombination component factors that contribute to capturing portable gene cassettes (Hall and Collis, 1995). In Enterobacteriaceae, the existence of class 1 integrons was highly linked with MDR (Kor et al., 2013).

The discovery in TET-resistant isolates of a high proportion of *tetA* and *tetB* genes indicates t the key TET resistance mechanism in isolated calf *E. coli* occurs by active efflux (Chirila et al., 2017). Among TET-doxycycline-resistant E, a predominance of the *tetB* gene was observed. Diarrheal *E. coli* isolated from calves correlated with prior results obtained in different countries (Chirila et al., 2017). Phenotypically susceptible strains of bacteria were carrying resistance genes, which may have contributed to their expression not yet occurring. Other experiments have also shown that certain bacteria lack the expression of resistance genes (O’Brien, 2002).

Integron genes are common in Enterobacteriaceae and contribute to MDR (Kang et al., 2005). Class 1 and class 2 integron genes were discovered in isolated *E. coli*. Their incidence was lower than that reported in *E. coli* isolated from poultry and pigs (Lapierre et al., 2008). Total 13 percent of *stx*-positive strains were also positive for integrons in the above study. A higher prevalence of integron-positive STEC strains isolated in the USA and derived from human patients (n=81) and domestic animals (n=193; Livestock) was observed in previous studies (Chirila et al., 2017).

Total 18 percent of 50 analyzed STEC strains originating from humans, livestock, and food (EFSA BIOHAZ Panel et al., 2020) Integron class 1 predominance reported strains possessed genes that coded for spectinomycin (*aadA1*) and trimethoprim resistance (*dfrA1*). Research conducted in Brazil on thirty-two STEC strains showed that in 22 percent of isolates, the integrase gene associated with Class 1 integrons, all of which had a uniform size and contained a single cassette gene (Chirila et al., 2017). Another aspect of all integron-positive strains showed factors of virulence that are important because of neonatal diarrhea. The strains of *E. coli* are a significant cause of economic losses on farms. These virulence factors draw attention not only to *E. coli* but also more resistant and more aggressive to antimicrobials.

## Treatment Strategies for Infections Caused by Shiga Toxin-Producing *Escherichia coli*

The inevitability of a STEC infection treatment strategy has a major issue in public safety and human health. Current treatment measure depends on hydration and antibiotic therapy (Ciccarelli et al., 2013). The frequent application of antimicrobial compounds towards the disease caused by STEC infections currently developed the risk of HUS association (Wong et al., 2000; Panos et al., 2006; Rahal et al., 2015).

## Novel and Alternative STEC Treatment Strategies

Different alternative therapies have been increased by a debatable use of antimicrobials in the management of STEC infection (**Table 1**). This ranges from the use of novel secondary metabolite towards different therapies that revisit antibiotics (Grisaru et al., 2017).

## Shiga Toxin Analog Receiver

Different drugs have been developed that imitate and bound Stx receptors, minimizing their accessibility to effector cells. Gb3-held carbosilane dendrimers deactivate Shiga toxins *in vitro* and have been shown to treat the impaired mice by intravenous administration (Jacobson et al., 2014). Conjugated carbohydrates-based compounds, such as STARFISH and DAISY, based on *in vitro* analysis, confirmed that the conjugated multivalent compound neutralizes Shiga toxins (Chabre and Roy, 2010; Bhatia et al., 2014). Highly clustered *Gb3* polymers bind with Shiga toxins with strong binding efficacy and treat the impaired mice when ingested orally (Tesh, 2010; Jacobson et al., 2014). Previous reports indicated that oral ingestion of synthetic version of verotoxin (*VT*, Shiga-like toxin) Pk-oligosaccharides-based receptor sequences attached to Chromosorb (Synsorb-Pk) by healthy adult volunteers. Sensor-based-Pk reclaimed from volunteer stool samples was also analyzed to determine if its *VT*-binding activity was affected by exposure to the pH extremes and digestive processes of the human gastrointestinal tract, but most of the compounds were found to be not providing promising results in the clinical studies (Armstrong et al., 1995).

## Shiga Toxins - Intracellular Interference

It has been stated that cell permutant agents are capable of binding Shiga toxin 2 (*Stx2*) and probably interfering with its traffic. The two agents were evaluated in animals, and *Stx2* inhibitor skills were demonstrated by both acetyl groups to all the amino termini of PPP-tet (yielding Ac-PPP-tet) (Rahal et al., 2015) and using baboons as an animal model, the cell-penetrating peptide (TVP) dock with *Stx2*, this leads to the reduction of toxin lethality with (55 ng/kg) (Stearns-Kurosawa et al., 2011) The intracellular transmission of the B subunits of *Stx* was also reported to interfere and defend against Stx1 and Stx 2 in Mice (Watanabe-Takahashi et al., 2010; Jandhyala et al., 2016). The *Retro-1* and *2* with small molecular inhibitors have also shown to be agents that interfere with *Stx* trafficking employing high-performance testing (Wahome et al., 2011; Abdelkafi et al., 2020).

## Antibody-Based Therapy

Antibodies have been identified that can bind and nullify the Shiga toxins effects (Reid and Burton, 2002), and monoclonal *anti-Stx* subunit A showed major benefits through invitro and *in vivo* (mouse) models. Anti-lipopolysaccharide anti corporations were shown to be protective (Yamagami et al., 2001; Mejías et al., 2016). An immunoglobulin-rich bovine colostrum preparation containing a high titer of anti-*Stx1* and antic *Stx 2* was also tested, and 13 patients and 14 placebo controls were compared with a colostrum-treated group. In colostrum-treated patients, the median level of stolen excretion was reduced but not significantly affected by the infection therapy presence in the subject’s stolen stools. This therapy’s effects on HUS development or other possible infection complications were not controlled by study subjects (Huppertz et al., 1999). The humanized monoclonal antibody Eculizumab in contrast to the complement component 5 (*C5*) has shown beneficial effects of STEC-associated HUS rehabilitation including clinical trials (Grisaru, 2014; Mahat et al., 2019).

## Natural Products (Secondary Metabolites)

Numerous metabolic products have been considered as possible STEC-based natural therapeutic drugs. Which include, in addition to plants, fruit and herbal products, grains and organic acid (Anand et al., 2019), and fruit drinks [Citrus limon (Rutaceae)] (Nogueira et al., 2003; Lacombe et al., 2010). These drugs were promising *in vitro* or *in vivo* (mice) models but were not tested in clinical trials. It is important to note a study showing a synergistic treatment effect with mice with STEC infection (Lee and Stein, 2011; Rahal et al., 2015; Ma et al., 2019) between processed tea leaves and an antibiotic, levofloxacin, which indicates that the significant threat of the treatment of antibiotics may decrease with the addition of another agent.

## Antimicrobial Drugs

The application in the treatment of STEC infections of antimicrobial agents was controversial and is under vigorous debate. Although certain studies showed that the ingestion of specific agents may increase the chance of Hemolytic Uremic Syndrome Risk (HUS), some observed a decline in this risk after antimicrobial application. Whereas some drugs may be specific at a certain dose, the significant threat of antibiotic therapy triggering HUS has led to a large contraindication of these agents (Safdar et al., 2002; Puño-Sarmiento et al., 2020). The threat of HUS by increasing Shiga toxins from the bacterial cells in a variety of ways is presumed to be increased by antimicrobial agents. The bacterial SOS response is the important signaling pathway for high-level production and release of *Stx1/2* prophages in STEC bacterial strain may be caused by the DNA damage which certain antimicrobials can cause. To activate the host DNA damage response pathway (SOS response), which is to cope with nucleic acid damage, leads to the production of different proteins that can encode an Stx and enhance its development by triggering the lytic cycle of the bacteriophages. Besides, some form of antibacterial agents stress may trigger lead to increased expression of toxins (Łoś et al., 2009; Kimmitt et al., 2000). In comparison, *Stx1* is known to be deposited in the cytoplasmic membrane of STEC strains, which can lead to increased liberation from cell lysis triggered by an antibacterial property in the specific Stx form (Vázquez-Laslop et al., 2006; Harms et al., 2016).

As with other prophages, the *Stx*-encoding prophets (e.g. quinolone antibiotics) induce by activating a host DNA damage reaction pathway (SOS reaction). Thus, quinolone antibiotics are associated with complications for EHEC infections. While transcriptional and translational inhibitors can demonstrate the possibility of inhibiting the Stx production, several studies indicate that antibiotic therapy raises the chances for EHEC-associated severe infection. It is not well explored the mechanism of *Stx1/2* expression can be blocked by SOS response (such as administration of quinolone). It was therefore attempted to decide whether antibiotics to stop Stx development of pre and post activation of the host SOS mechanism that suppresses bacterial toxin gene expression can be applied (Zhang et al., 2016).

Nevertheless, in recent decades, has been developed an interest in the management of STEC-based infections with antibacterial drugs. The threat of HUS (caused STEC *E. coli*) subjects has subsequently been decreased by ciprofloxacin and subjects treated with azithromycin were also observed during the 2011 outbreak (Freedman et al., 2016; Berger et al., 2019). It was evaluated that the use of rifampicin decreases the toxin synthesis, but with *E. coli* O157 serotype appears to be sustainable, followed by gentamicin treatment at a lethal stage of infection. Especially in comparison with the bactericidal gentamicin dose, this technique was effective in reducing the production of toxins (McGannon et al., 2010; Puño-Sarmiento et al., 2020). A similar approach should be applied in an *E. coli* O157:H7 mice inflammation model that contributed to a higher survival rate for animals (Rahal et al., 2012; Fadlallah, 2014; Rahal et al., 2015). The findings indicated that various STEC serotypes significantly respond to the therapy of antimicrobial agents.

## Phage Based Prevention

The application of lytic phages is another preventive measure suggested as a way to monitor STEC. Lytic phages have shown that they may be reduced by the amount of STECs *in vitro* (Rivas et al., 2010; Chen et al., 2020). Phage-containing materials for the management of STEC species can be spread in animal fur or processed meats, are commercially available, and are licensed by the Food and Drugs Administration (FDA) (Anany et al., 2011a; Anany et al., 2011b; Tolen, 2018; Endersen and Coffey, 2020). Nevertheless, the effectiveness of oral treatment on domesticated animals with lytic phages was shown to be effective, and an improved method or delivery method is required (Pinto et al., 2020). In the case of human safety and effectiveness, the application of phages acts as a therapeutic utility against STEC *E. coli*.

Numerous vaccine-based scenarios have been attempted to establish the antimicrobial strategies that include bacterial secondary metabolite-based peptides and virulence factors like (Jamalludeen, 2006; Nonis, 2016; Tolen, 2018; Akindolire, 2019). These vaccine formulations have been tested with some positive findings in mice models (Garcia-Angulo et al., 2013).

## DNA Based Vaccines Towards Prevention of STEC

Antibiotic treatment of STEC-infected patients increases the incidence of infection rather than amelioration, possibly due to cell wall damage of STEC *E. coli* and the liberation of more Shiga toxins. Consequently, there remains a need to develop a technique to generate antibodies against *E. coli* to prevent and alleviate STEC infections. Hence, vaccination is considered an appealing strategy to reduce STEC colonization. Vaccines are substances that interact with the immune system to trigger antibodies’ production, which subsequently provides immunity against serious, life-threatening diseases (Nabel, 2013; Greenwood, 2014; Gómez and Oñate, 2018). The notion of vaccination was revealed 200 years ago when Jenner demonstrated that former acquaintance to cowpox could avert infection by smallpox (Gurunathan et al., 2000).

DNA vaccines comprise a bacterial plasmid with a robust viral promoter, the gene of interest, and a transcriptional stop sequence (Snedeker et al., 2012; Zhang and Sack, 2015). The genetically engineered DNA-based vaccine is taken up by host cells where the encoded protein is made. Recent research has explored the prevention of STEC infections using DNA-based vaccines (Bourgeois et al., 2016; Harding and Feldman, 2019; Jeshvaghani et al., 2019; Rodrigues-Jesus et al., 2019). Animal sources are one of the main reservoirs for STEC and a leading cause of STEC infections in cattle. Vaccination is a promising strategy to reduce the prevalence of STEC in cattle and significantly reduce the incidence of disease in humans (Cox et al., 2014; Saeedi et al., 2017). Four immunologically significant genes, *stx, espA, eae*, and *tir* are the leading DNA vaccine candidates to prevent STEC infections (**Supplementary Table 7**). Many researchers have reported on DNA-based vaccines targeting these leading candidates (Bentancor et al., 2009; Cai et al., 2011; Gu et al., 2011; Mehr et al., 2012). Gao et al. (2009) described DNA vaccination targeting *stx*_1_ and *stx*_2_ using the mouse model. A novel fusion protein was developed and induced a high level of humoral IgG in mice. This fusion protein elicits a high level of neutralizing antibodies and protected mice from the lethal dose challenge of STEC. Mehr et al. (2012) generated a protective immune response against EHEC by producing antibodies targeting *Esp*A and *Tir* proteins. DNA vaccine could induce protective immunity in BALB/c mice against *E. coli* O157:H7. A DNA vaccine has been reported in another study that targets *stx*, *esp*A, and intimin virulence factors. The vaccine-induced a strong humoral response and protected mice against infection with live EHEC or EHEC sonicated lysate. Besides, the vaccine enhanced evacuation of gut colonized *E. coli* (Gu et al., 2009). Meanwhile, many of the alternative virulence factors such as *EspB, EspD, NleA, TccP*, and *NleB* of EHEC have attracted vaccine designers’ attention (Creuzburg and Schmidt, 2007; Roe et al., 2007; Misyurina et al., 2010). Asper et al. (2011) recently constructed a vaccine generating antibodies against *EspB, EspD*, *NleA*, and *EspA*. These factors contributed to the future candidates for the treatment of STEC infections.

DNA vaccination is a novel, economic, and effective strategy to prevent various infectious diseases, with additional advantages over live attenuated bacteria including the ease of design and construction, low cost, safety, and long-lived responses (Creuzburg and Schmidt, 2007; Roe et al., 2007; Gao et al., 2009; Gu et al., 2009; Fioretti et al., 2010; Misyurina et al., 2010; Asper et al., 2011; Mehr et al., 2012). Furthermore, DNA vaccines are a promising strategy to decrease STEC infection and spread in animals, the environment, and eventually humans. However, many challenges remain in developing a vaccine for humans. The success of a DNA vaccine depends on the nature of the host to be immunized, optimization of the DNA vaccine, an appropriate choice of a plasmid vector, and the type of immune response generated (Smith et al., 2006; Tsuji et al., 2008; Cai et al., 2011).

## Current Scenario of Probiotic Therapy in Eradiation of STEC Towards a Replacement of Antibiotic Therapy

**Figure 3:**
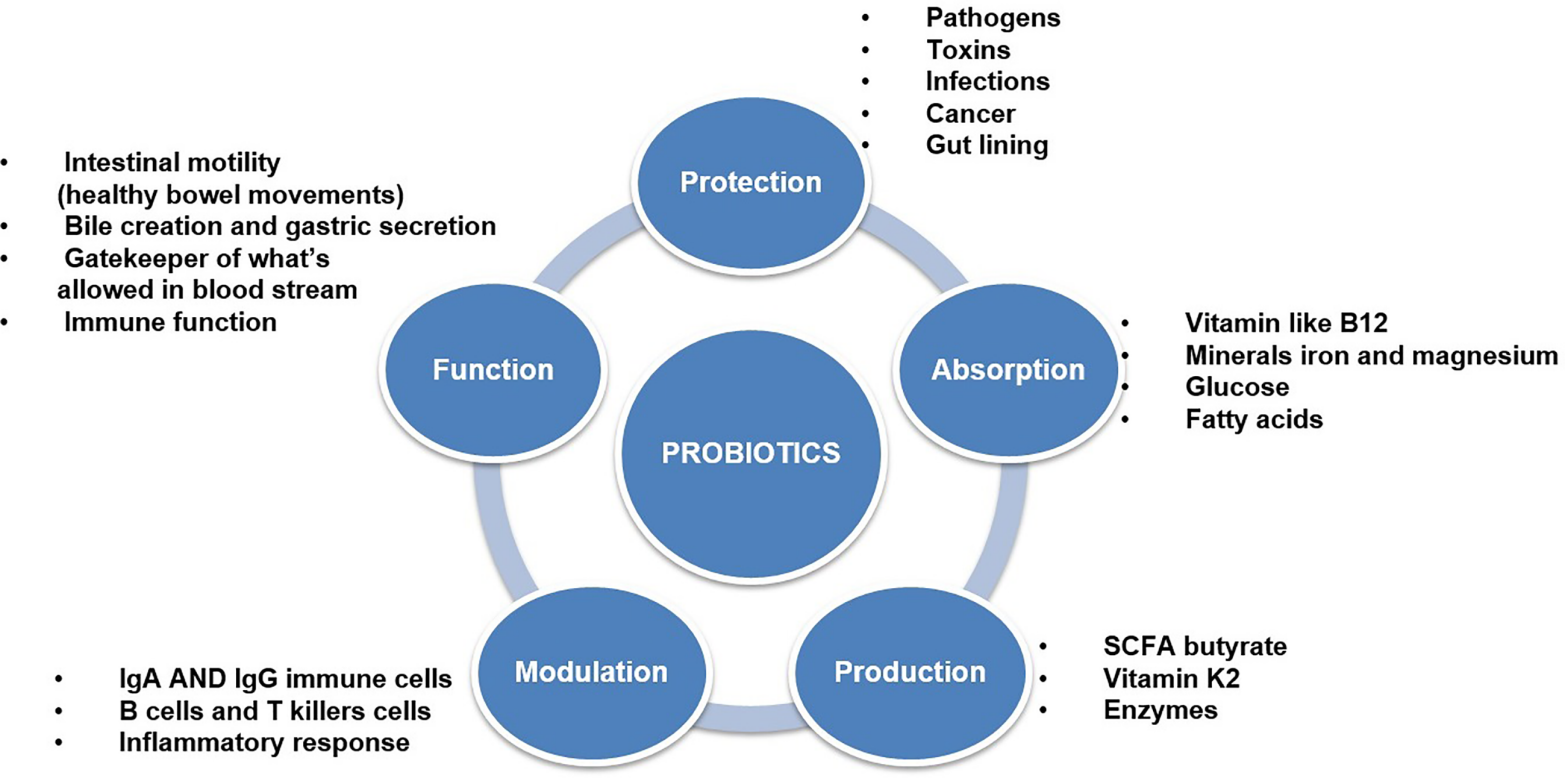
Efficacy of probiotics and different types of functional properties.

Lactic Acid Bacteria (LAB) are Gram-positive, non-motile, non-spore-forming, facultative, or obligate anaerobes with a spherical or rod-like shape. LAB can stimulate numerous immune responses by distressing specific receptors in the host’s gut or immune cells (Bene et al., 2017; Kim et al., 2017). Activation of these receptors leads to the production of widely used effectors, such as cytokines or T cells (Frederick et al., 2017). These LAB, which have a beneficial effect on health, are often referred to as probiotics. Probiotics are live microbes which promoted for health benefits and help to restore the microbiota. Probiotics are safe to consume but the specific functional metabolic activity has to be determined for a particular health effect (Georgalaki et al., 2017) (**Figure 3**). Effective probiotics that are nonpathogenic and non-toxic were also needed to be proficient in exerting a beneficial effect on the host. Probiotic organisms can help control pathogenic bacteria by producing a wide range of antimicrobial substances such as acidophil, bacitracin, bacteriocin and some minor short-chain fatty acid, which binds with the bacterial cell wall-based lipoprotein leads to damage the cell wall or it may bind with the topoisomerase enzyme, which intends to inhibit the replication process further it may inhibit the protein synthesis, further some of the polyphenolic compounds such as quaracitin or tannin compounds which may also act as an inhibitory compound. The Lactic acid produced by probiotics decreases the pH of the host intestine and inhibits bacterial pathogens such as the genera *Escherichia*, *Clostridium*, *Salmonella*, and *Shigella*. Furthermore, effective probiotic organisms reduce the production of various toxic or carcinogenic metabolites and competitively block adhesion sites of intestinal epithelium inside the host (Singh et al., 2013; Rodjan et al., 2018) (**Supplementary Table 8**).

## Effect of Probiotic Yeast Therapy Against STEC

Yeasts are eukaryotic microbes widely found in natural environments, such as animal microbial flora, soil, plants, water, airborne particles, food, and other niches (Kurtzman et al., 2011; Hatoum et al., 2012). Yeasts play a significant role in complex ecosystems (Möndel et al., 2009). They also interact with numerous defined microorganisms in a range of processes including mutualism, symbiosis, parasitism, and antagonism. Yeasts are a significant component of the micro-flora of different fermented foods and beverages; the yeast habitat of human and animal origins has a significant impact on their food safety and nutritive features. Brewer’s yeasts (*Saccharomyces* spp.) are most commonly available as dietary supplements due to their enhanced nutritional and mineral content. Despite their non-human origin, such non-pathogenic yeasts fulfill the key criteria for probiotic definition as follows; the World Health Organization defines probiotics as “live microorganisms which when administered in adequate amounts confer a health benefit on the host” (WHO, 2002); to be labeled probiotic, scientific evidence for the health benefit would have to be documented. The most common probiotics are Gram-positive LAB of the genera *Lactobacillus sensu lato* and *Bifidobacterium*, but yeasts such as *Saccharomyces boulardii* (Terciolo et al., 2019) are also used as dietary supplements or as a pharmaceutical aid for therapeutic agents (Möndel et al., 2009). *S. boulardii* is non-pathogenic yeast that has been used internationally and extensively as a probiotic since the 1950s. Lyophilized yeast (*S. boulardii*) is available as a dietary supplement for children and adults in 250 mg capsules and may be prescribed as 1–2 capsules to be taken 1–2×/day by Biocodex, USA. The product package shows the following claims for structure/function: a) retains the intestinal flora balance, b) keeps the intestines working well, and c) helps intestinal health. Using a gnotobiotic mouse model of *Shigella flexneri* infection, (Rodrigues et al., 2000) proved that *S. boulardii* cultures protected mice from pathogen-associated tissue harm without lowering the levels of *Shigella flexneri* in the bowel. The direct impacts of *S. boulardii* on the intestinal mucosa, specifically the stimulus of enzymatic activity and the enhancement of the host’s intestinal mucosal immune response, are the likely mechanisms by which *S. boulardii* protects the host from diarrheal pathogens (Barc et al., 2008). *S. boulardii* differs significantly from *S. cerevisiae* in metabolic and physiological terms, especially in terms of growth yield and resistance to temperature and acid stress. While the majority of strains of *S. cerevisiae* grows and metabolize at 30°C, *S. boulardii* acts as thermophilus yeast, growing at 37°C, the physiological temperature of the host. Recent research showed that *S. boulardii* was much more resistant to a simulated gastrointestinal condition than *S. cerevisiae* strain W303 (Tiago et al., 2015). The improvement of microbial diversity in the intestines is one of the main modes of action for *S. boulardii* as demonstrated by the use of monogastric experimental designs for improved intestinal health (Möndel et al., 2009).

Interest in probiotic yeast has been raised predominantly in domestic animal feed preparation, and human applications because yeasts are rarely correlated with food-borne illness. Based on their history, most yeast species are recognized as safe by the European Food Safety Authority (EFSA, 2014). Studies reported that some *S. boulardii* strains originally selected using empirical methods, can act as an antidote against various gastrointestinal diseases (Buts, 2009; Vandenplas et al., 2009), hence *S. boulardii* is recognized as prototype of non-bacterial probiotics. Several mechanisms have been suggested for the broad health-promoting effects of consuming food-grade yeasts (Czerucka and Rampal, 2002; Czerucka et al., 2007). Some of the reported effects of yeasts as probiotic organisms in clinical trials are (i) Antibiotic-associated diarrhea; (ii) Infectious diarrhea (including that caused by recurrent *Clostridium difficile* infection); (iii) irritable bowel syndrome; and (iv) Inflammatory bowel diseases (IBD) (Foligne J. et al., 2010). *S. boulardii* efficacy was both in preventing and treating diarrhea and colitis in humans associated with antibiotics (Guslandi et al., 2003).

## Effect of Prebiotics (Oligosaccharides) With Synbiotic Activity Towards Reduction of STEC Infection

The pectic-based oligosaccharide from the plant-based origin has been previously reported to control STEC pathogens; the pectin consists of homogalacturonan as a backbone and arabino- galacto oligosaccharide, which is enzymatically treated and methylated and protects the human colonic HT29 cells from the Shiga toxin-producing *E. coli* at 10mg/mL. Previous reports suggest that galacturonic acid disaccharides supported the anti-adhesion activity and trisaccharides against *E. coli*; further, oligosaccharides’ concentration was mainly correlated with the anti-adhesion activity. Likewise, a study conducted by Hotchkiss (2015). Indicated that Xyloglucon extracted from the cranberry act as an effective inhibitory to the adhesion of STEC strain in the HT29 cells at a lower concentration, and it was also concluded that the adhesion mechanism was mainly due to the fimbriated *E. coli*. Pectin is extracted from the root of Panax ginseng, primarily consisting of galacturonic and glucuronic acids with rhamnose, arabinose, and galactose present as minor components exerted selective anti-adhesive effect against pathogenic bacteria *E. coli* and *Staphylococcus aureus*.

**Figure 4:**
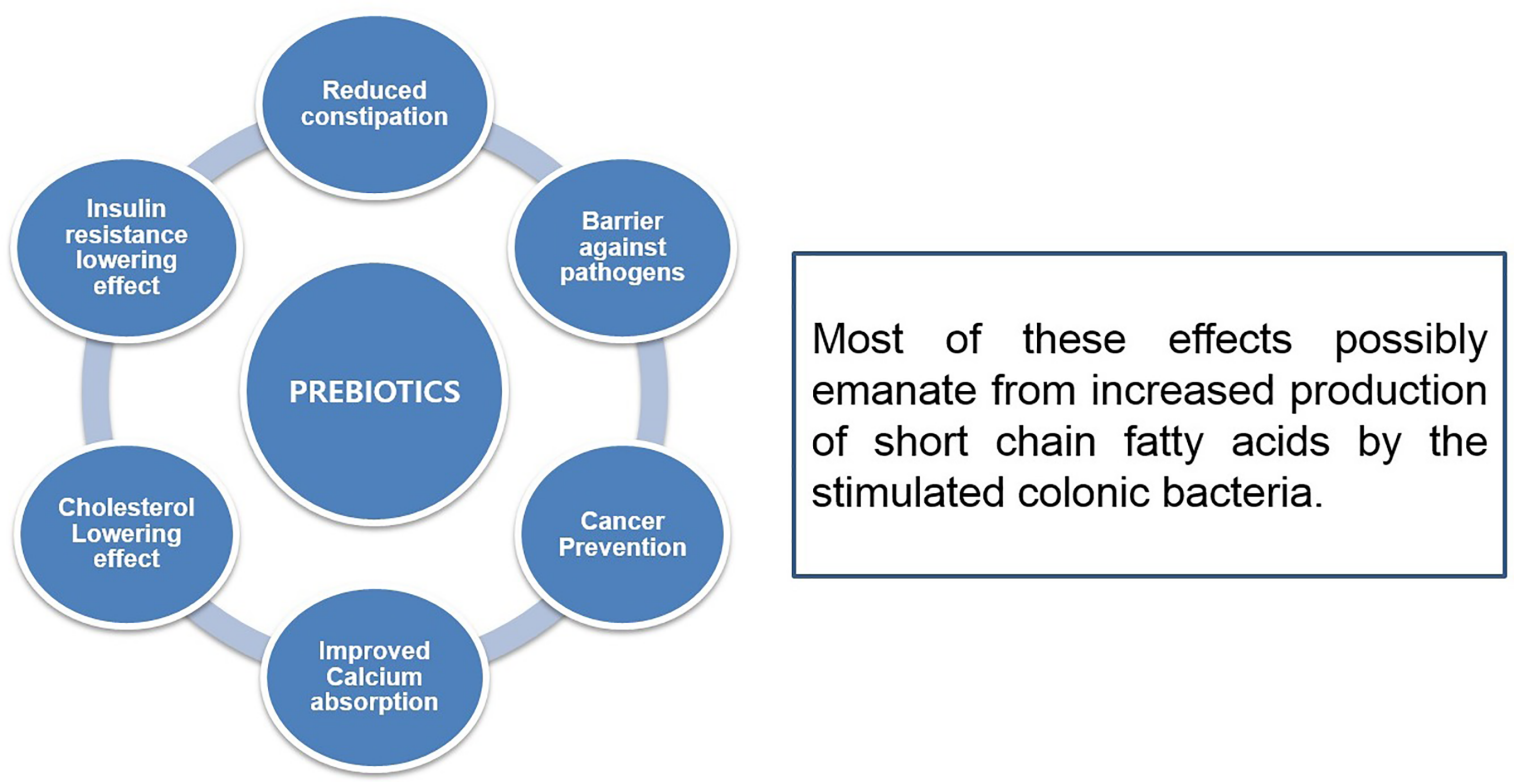
Efficacy of prebiotics and different types of functional properties.

**Figure 5:**
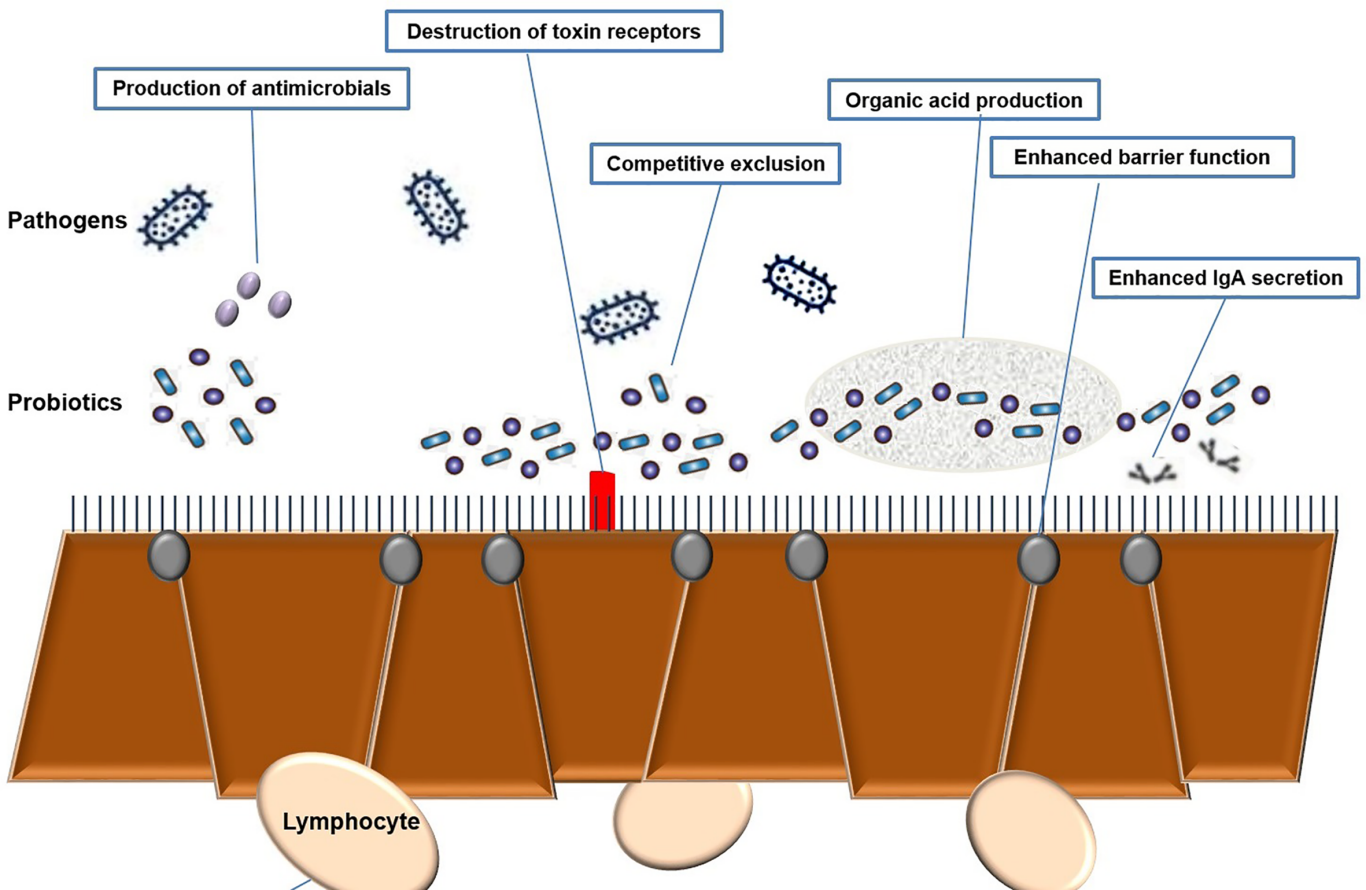
Mechanism of probiotics towards host-pathogen [Shiga toxin-producing *Escherichia coli*, Enterohemorrhagic *Escherichia coli* (EHEC)] interaction.

**Figure 6:**
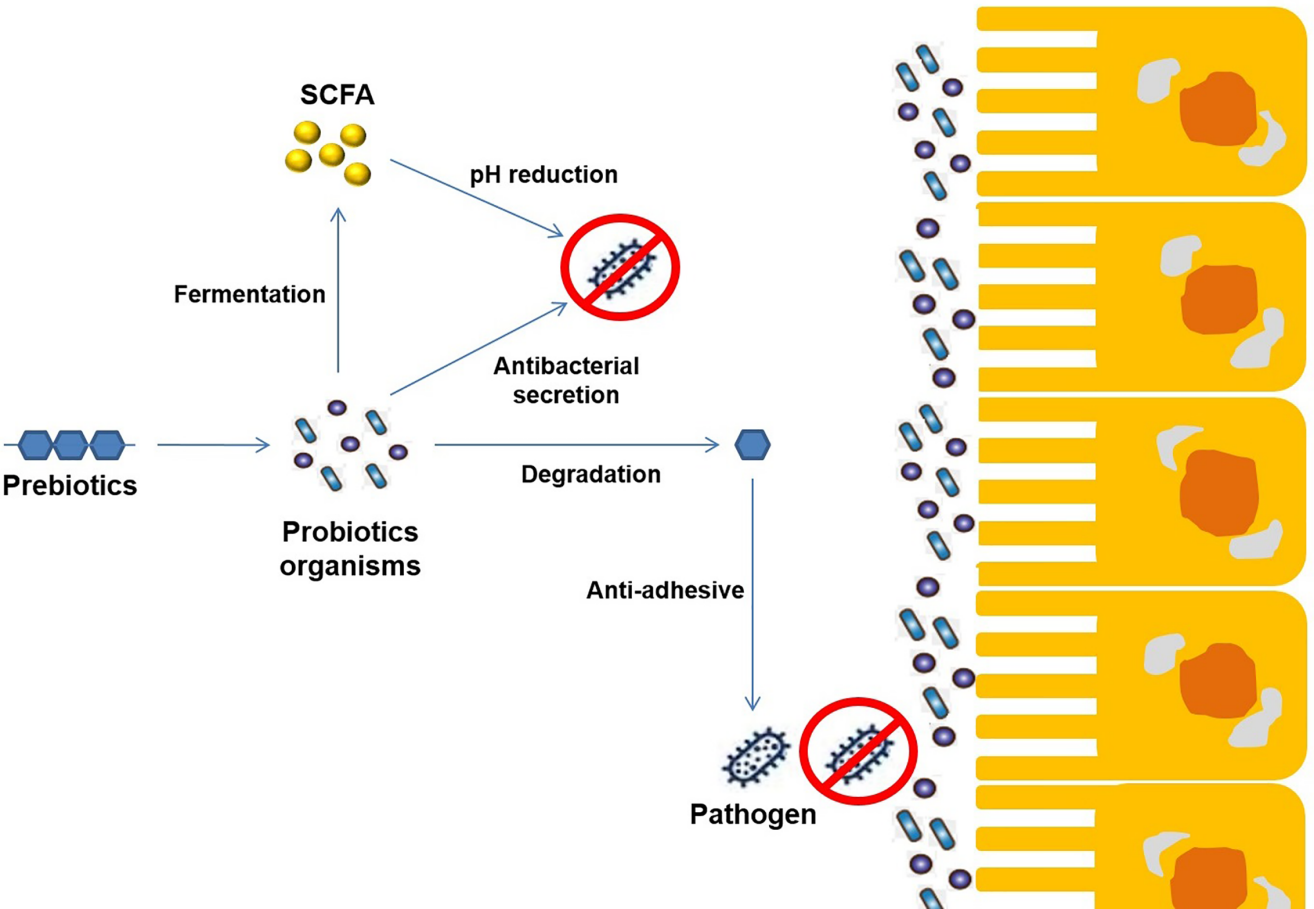
Schematic representation of the mechanism of synergetic activity (Probiotics+ Prebiotics) towards different enteropathogenic infections.

Prebiotic oligosaccharides, including FOS, XOS, and GOS, are classified as non-digestible dietary ingredients that benefit the host gastrointestinal tract (**Figure 4**). Initial non-intimate adherence is therefore an essential aspect of STEC pathogenesis because it is the first infection stage. Preventing this first adherence step will eventually hinder the cycle of infection. Oligosaccharides can stimulate the growth of beneficial intestinal microbial groups such as *Lactobacillus* spp. and *Bifidobacterium* spp., reduce constipation, and decrease colon cancer risk, promote immune-stimulation in the intestinal tract, and improve the function and health of the intestinal tract (**Figure 5**). Some intestinal pathogens, such as STEC, express multifarious proteins that allow them to adhere to separate receptor sites of oligosaccharides located on the host cell surface (Baker et al., 2016; Saeedi et al., 2017; Valilis et al., 2018). FOS is composed of glucose and fructose molecules linked by a degree of polymerization (DP) of 2-9 (DP is the number of glucose and fructose molecules) (Soleimani et al., 2012). Detailed investigations have demonstrated that oligosaccharides have functional effects in lipidemia and cholesterol decreases (Manosroi et al., 2014), inhibition of aberrant crypt foci formation, prevention of osteoporosis due to increased bone strength, inhibition of diarrhea (Akrami et al., 2013), and reduction of the risk of atherosclerotic cardiovascular disease (Ahmed, 2014). Different oligosaccharides have also shown anti-adhesive activity. Dietary oligosaccharides have been isolated from natural sources such as food grains, agricultural waste products, human breast milk, while others have been synthesized based on the known components of glycolipid oligosaccharides and glycoproteins that border the gastrointestinal tract cell surface (Baker et al., 2016). Numerous reports support the role of glycolipids, glycoproteins, and soluble oligosaccharides as molecular decoys to host cell surface oligosaccharides (**Figure 6**).

Probiotics based on beneficial microbial strains have additional health benefits (Chelliah et al., 2018). Some probiotics are effective against specific toxin-producing enteropathogens such as emetic toxin-producing *Bacillus cereus*, STEC, enterohemorrhagic *E. coli* (EHEC), and enteropathogenic *E. coli* (EPEC), which cause urinary tract infections (UTIs) and gastrointestinal infections (Hwang et al., 2018). Most probiotics were found in the gut microbiota, which mainly originates from our food intake and lifestyle of fermentation-based food products, dairy-related products, and traditional food products based on cereals and pulses (McLellan and Hunstad, 2016; Chelliah et al., 2018). The probiotics vary based on their specificity on functional activity (Gibson et al., 2017). Indirectly specific prebiotic compounds (galactooligosaccharide – GOS, fructooligosaccharide – FOS, xylooligosaccharide XOS, and inulin) encompassed functional food was mainly responsible for the gut microbiota modulation in which the phylum *Bacteroidetes* were enhanced while there was a corresponding decrease in the phylum *Firmicutes* (Chaparro, 2017; Castaner et al., 2018; Collado et al., 2019).

## Conclusion

Among global foodborne bacterial pathogen outbreaks, the main cause of Gastroenteritis in adults and children is STEC infection. Despite the key improvements in sympathetic of STEC mechanism, no explicit effective management is presently available. The consolidated results in the review open a novel concept towards controlling the STEC infection. Further, based on the *in-vivo* and *in-vitro* data, clinical trial in humans helps us to determine the efficiency of symbiotic treatment (Probiotic+ prebiotics) and a simple cost-efficient reliable methodology were determined to understand and to differentiate the mechanism of STEC and non-STEC infection. Further, a similar methodology can be applied to understand host-pathogen interaction. To sum up, a widely accepted effective therapeutic procedure for the species remains undocumented, despite more than five decades after STEC strain was initially identified with clinical studies. Fortunately, a variety of methods have been pursued, including those to rethink the application of antimicrobial agents; benefits to certain agents, findings with antimicrobial-based results, their dose, and STEC itself, have been recorded. Additional tests of antimicrobial agents for the therapy of infection with STEC in animals should be carried out to select the best and most effective diet to be tested in the clinical trials.

## Author Contributions

The manuscript was written in detail and sectioned for specialized discussion with the respective authors in the field of research. Designing the outline of the Review manuscript [Shiga-toxin producing *E. coli* (STEC) Gastroenteritis management: Is there a role for Probiotics?: A Systematic Review], Visualization, Conceptualization – (S-bH, RC, D-HO). Mode of transmission of *Escherichia coli* (STEC) and the effect of shiga toxin in humans and animals– JK. 2. Survival efficacy of *Escherichia coli* (STEC) in the intestine environment, Correlation of antimicrobial resistance towards increased toxin gene expression of *Escherichia coli* (STEC) – RC, EB-M. DNA vaccines towards prevention of *Escherichia coli* (STEC), Toxic Effect of *Escherichia coli* (STEC) - *In-vivo* model (Caenorhabditis elegans), Current scenario of probiotic therapy in eradiation of *Escherichia coli* (STEC) towards replacing of antibiotic therapy – RC, S-bH. Effect of probiotic (yeast) therapy against *Escherichia coli* (STEC), Effect of probiotics against *Escherichia coli* (STEC) - *In-vivo* model (Caenorhabditis elegans), Prebiotic based oligosaccharides reduce adherence of enteropathogenic *Escherichia coli* (STEC) – FE, RC. All authors contributed to the article and approved the submitted version. First Author: S-bH, RC (Equal Contribution). *Corresponding author: D-HO (deoghwa@kangwon.ac.kr) *Co-Corresponding author: RC (ramachandran865@gmail.com).

## Funding

The Article Processing Charges have been covered by Korea Research Fellowship Program through the National Research Foundation of Korea (NRF) funded by the Ministry of Science, in Young Researchers Program [2018007551].

## Conflict of Interest

The authors declare that the research was conducted in the absence of any commercial or financial relationships that could be construed as a potential conflict of interest.
