# Supplementary figures and images for: Role of Recent Therapeutic Applications and the Infection Strategies of Shiga Toxin-Producing Escherichia coli
Source: Front Cell Infect Microbiol. 2021 Jun 29;11:614963. doi: 10.3389/fcimb.2021.614963 (PMC8276698; doi:10.3389/fcimb.2021.614963)

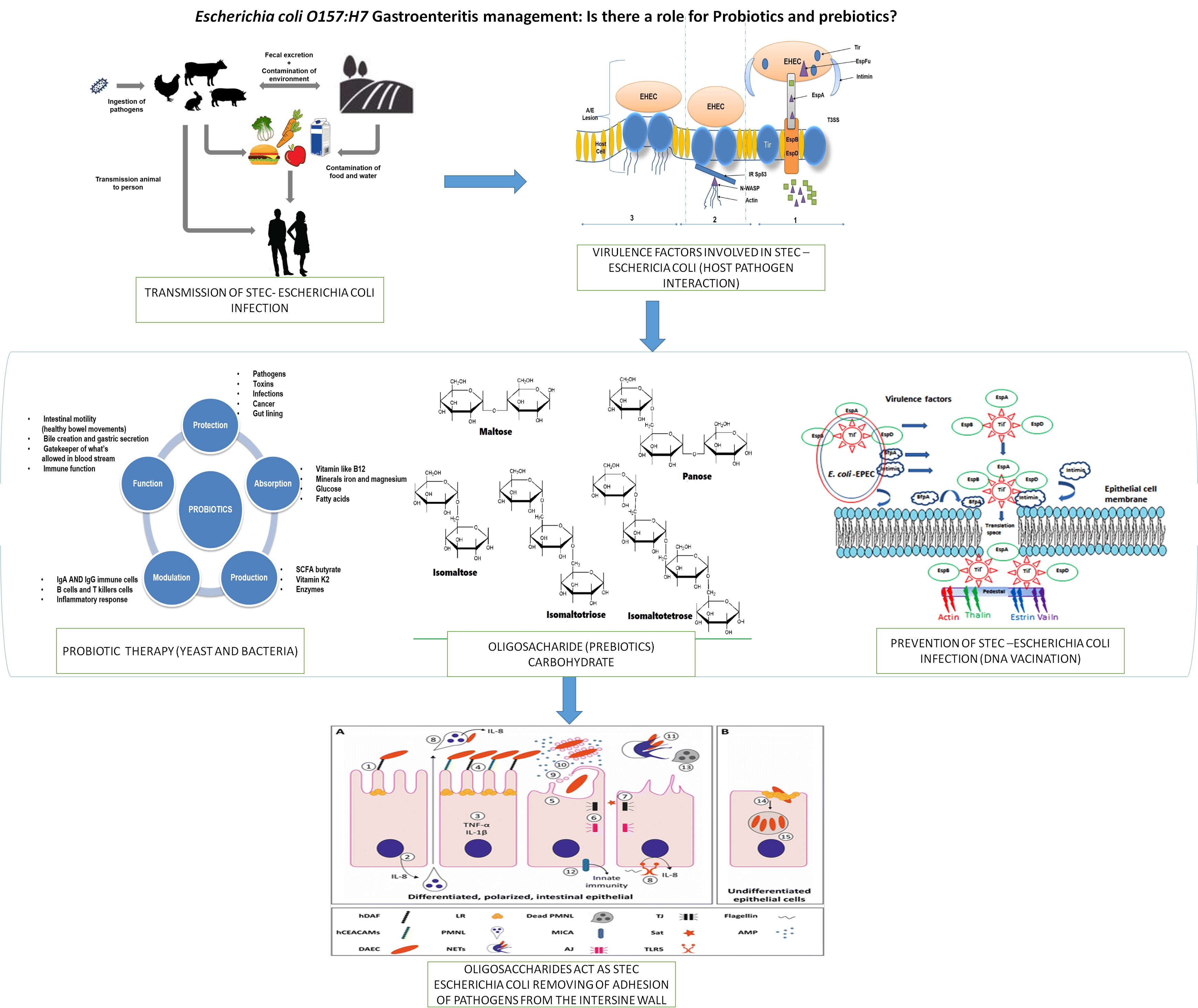

Supplement: Supplementary file 1 [file Image_1.jpeg]
