# Supplementary material for: Role of Recent Therapeutic Applications and the Infection Strategies of Shiga Toxin-Producing Escherichia coli
Source: Front Cell Infect Microbiol. 2021 Jun 29;11:614963. doi: 10.3389/fcimb.2021.614963 (PMC8276698; doi:10.3389/fcimb.2021.614963)
Supplement: Supplementary file 2 [file Table_1.doc]

**Supplementary Table 1.** Non-outbreak reports of non-O157 STEC strains by global region, serogroup, number reported, and numbers with dysentery and hemolytic uremic syndrome of those observed for these complications, 1995–2017. (Copy right obtained from Valilis et al., 2018)

| **Regions** | **Serogroups/types (number reported)** | **Number of reported cases of HUS (%)** | **Number of reported cases of dysentery (%)** | **Number reported** |
| --- | --- | --- | --- | --- |
| USA and Canada | O26 (292), O45 (211), O111 (201), O103 (207), O121 (110), O145 (75), other serogroups/types (416) | 45/1460 (3.1%) | 432/1454 (30%) | 1512 |
| Europe | O26 (216), O63 (11), O103 (71), O111 (54), O145 (51), O146 (20), other serogroups/types (103) | 60/512 (12%) | 95/512 (19%) | 526 |
| South and Central America | O26 (107), O103 (43), O111 (52), O145 (55), O146 (10), O174 (3), other serogroups/types (297) | 119/567 (21%) | 38/481 (7.9%) | 567 |
| Australia | O26 (7), O103 (1), O111 (14), O113 (1), O172 (1), other serogroups/types (45) | 7/69 (10%) | 58/67 (87%) | 69 |
| Japan | O65 (5), O103 (13), O111 (15), O121 (7), O145 (3), O165 (5), other serogroups/types (26) | 4/74 (5.4%) | 23/74 (31%) | 74 |
| Total |  | 234/2682 (8.7%) | 646/2588 (25.0%) | 2748 |

HUS, hemolytic uremic syndrome; STEC, Shiga toxin-producing *E. col*
