# Supplementary material for: Role of Recent Therapeutic Applications and the Infection Strategies of Shiga Toxin-Producing Escherichia coli
Source: Front Cell Infect Microbiol. 2021 Jun 29;11:614963. doi: 10.3389/fcimb.2021.614963 (PMC8276698; doi:10.3389/fcimb.2021.614963)
Supplement: Supplementary file 3 [file Table_2.doc]

**Supplementary Table 2.** Timeline of outbreaks, recalls, and government response with relation to STEC. (Copy right obtained from Baker et al., 2016)

| **Year** | **Summary** | **Reference** |
| --- | --- | --- |
| 1977 | Cytotoxicity of *E. coli* is first observed | Konowalchuk, Speirs, & Starvic, 1977 |
| 1982 | Food-borne outbreaks led to the identification of *E. coli* O157:H7 | Riley et al., 1983 |
| 1993 | Washington state outbreak (including Idaho, Nevada, and California) | Bell et al., 1994 |
| 1994 | FSIS declares *E. coli* O157 to be an adulterant in raw non-intact beef | Schuller, 1998 |
| 1996 | *E. coli* O157:H7 outbreak in Sakai City, Japan – over 7000 children linked to radish sprouts in school lunches | Sobel et al., 2002 |
| 1997 | Colorado recall of 25.6 million pounds. of frozen ground beef patties due to *E. coli* O157:H7 | CDC, 2007a |
| 2002 | Colorado recall of 18.6 million pounds of ground beef products due to *E. coli* O157:H7 | Vogt & Dippold, 2005 |
| 2006 | U. S. outbreak of *E. coli* O157:H7 linked to spinach | Grant et al., 2008 |
| 2011 | FSIS declares *E. coli* O26, O45, O103, O111, O121 & O145 to be adulterants in raw non-intact beef | FSIS, 2011 |
