# Supplementary material for: Role of Recent Therapeutic Applications and the Infection Strategies of Shiga Toxin-Producing Escherichia coli
Source: Front Cell Infect Microbiol. 2021 Jun 29;11:614963. doi: 10.3389/fcimb.2021.614963 (PMC8276698; doi:10.3389/fcimb.2021.614963)
Supplement: Supplementary file 4 [file Table_3.doc]

**Supplementary Table 3.** Illness and cost estimates of *E. coli* and other common pathogens in the U. S. annually. (Copy right obtained from Baker et al., 2016)

| **Pathogen** | **Illnesses** | **[Cost (millions ($))b](https://www.sciencedirect.com/science/article/pii/S0956713515300402#tbl1fnb)** |
| --- | --- | --- |
| Norovirus | 5,461,731 | 3677 |
| *Salmonella* (nontyphoidal) | 1,027,561 | 11,391 |
| *Staphylococcus aureus* | 241,148 | 168 |
| STEC non-O157 | 112,752 | 154 |
| STEC O157 | 63,153 | 635 |
| Non shiga-toxin producing *E. coli* | 29,876 | 40 |

a - Scallan et al., 2011.

b Scharff, 2012, cost estimate model accounts for pain, suffering and disability.
