# Supplementary material for: Role of Recent Therapeutic Applications and the Infection Strategies of Shiga Toxin-Producing Escherichia coli
Source: Front Cell Infect Microbiol. 2021 Jun 29;11:614963. doi: 10.3389/fcimb.2021.614963 (PMC8276698; doi:10.3389/fcimb.2021.614963)
Supplement: Supplementary file 5 [file Table_4.doc]

**Supplementary Table 4.** Summarized approaches of EHEC vaccination. (Copy right obtained from Saeedi et al., 2017)

| **Candidate antigen(s)** | **Administration Route** | **Immune responses** | **Result (Mean Survival)** | **References** |
| --- | --- | --- | --- | --- |
| Stx1or Stx2/liposome | Intra-venous | Anti Stx1/Stx2 | 80% | Suzaki et al 2002;  Uchida 2003; |
| Stx1B/Stx2B | Intra-peritoneal | Anti Stx1/Stx2 | 60% | Gao et al 2009 |
|  | Intra-nasal |  | 50% | Tsuji et al 2008 |
| Stx2A/Stx1B | Intra-peritoneal | Anti Stx1, Stx2/Th2- Response | 95% | Cai et al 201;  Smith et al 2006 |
| Mutated Stx2A & Stx2B | Intra-gastrically | Anti Stx2 & Stx2B/IgG | ∼20% | Bentancor et al 2010 |
|  | Intra- muscular | Anti Stx2 & Stx2B/IgG | 60% |  |
| EspA/intimin/Stx2B (EIS) | Subcutaneous | Anti EspA, intimin and Stx2 IgG | 93% | Gu et al 2009;  Gu et al 2011 |
|  | Intra-gastrically | Anti EspA, intimin and Stx2 IgG & IgA | 75% |  |
| EspA/intimin/Tir (EIT) | Subcutaneous | Anti EspA, intimin and Tir IgG | 90%, | Amani et al 2010;  Amani et al 2011 |
|  |  |  | ↓shedding |  |
| EspA/Intimin (EI) | Subcutaneous | Anti EspA and Intimin IgG | 70% | Rad et al 2013 |
|  |  | strong humoral response | ↓shedding |  |
| Intimin/Tir (IT) | Subcutaneous | Anti Intimin and Tir IgG | 68% | Yazdanparast et al., 2012 |
|  |  | strong humoral response | ↓shedding |  |
| EspA-Stx2A1 | Subcutaneous | Anti EspA and Stx2 IgG | 95% | Cheng et al 2009 |
| Stx2-Tir-Stx1B-Zot | Subcutaneous | Anti Tir, Stx1 and Stx2 IgG | 50% , | Zhang X-h et al 2011 |
|  |  |  | ↓shedding |  |
| H7-HCP-Tir-Intimin | Intravenous | Anti H7-HCP-Tir-Intimin IgG & IgA | ↓shedding | Zhang, Z. Yu,  Zhang, K. He., 2014 |
| Stx2B-Stx1B-Intimin (SSI) | Intra-peritoneal | Anti Stx1and Stx2 IgG | 60% | Gao et al 2011 |
| K-alpha-Stx2B | Intra-peritoneal | IgG & sIgA | 60% | Fujii et al 2012 |
| KT-12 intimin B-cell epitope | Subcutaneous | IgG & sIgA | 60% | Wan et al 2011 |
|  | Intra-nasal | IgG | 27% |  |
| Type III-secreted proteins (TTSPs) | Subcutaneous | Anti TTSPs IgG | 100% | Babiuk et al 2008; A.A. Potter et al 2004 |
|  | Intra-nasal | Anti TTSPs IgG & IgA | 60% |  |
| Intimin | Intra-gastrically | IgG & sIgA | ↓shedding | Fan et al 2012;  McNeilly et al 2010 |
| Tir | Subcutaneous | IgG | 65% |  |
|  | Intra-nasal | IgG & sIgA | 93% |  |
| ler/stx1 or 2 | Intra-peritoneal |  | 60% , | Liu et al 2009 |
|  |  |  | ↓shedding |  |
| BG | Intra-gastrically | IgG & sIgA | 70% , | Yamasaki 2002 |
|  |  |  | ↓shedding |  |
|  | Rectal | IgG & sIgA | 100% , |  |
|  |  |  | ↓shedding |  |
| EIT plasmid DNA vaccine | Intramuscular | IgG | ↓shedding | Shariati Mehr et al 2012 |
| SRP | Oral | anti-SRP antibodies | 77% , | Cull et al 2012;  Fox et al 2009;  Sharma et al 2011;  Thornton et al 2009 |
|  |  |  | ↓shedding |  |
|  | p arenteral | humoral immune response | 63% , |  |
|  |  |  | ↓shedding |  |
| PNAG (9GlcNH2)/Stx1B | Intra-peritoneal | Anti Stx1 & Stx2 IgG | 100% | X. Lu et al 2014 |

Abbreviations: BG., Bacterial Ghosts; SRP, siderophore receptor and porin proteins; PNAG, poly-N-acetylglucosamine
