# Supplementary material for: Role of Recent Therapeutic Applications and the Infection Strategies of Shiga Toxin-Producing Escherichia coli
Source: Front Cell Infect Microbiol. 2021 Jun 29;11:614963. doi: 10.3389/fcimb.2021.614963 (PMC8276698; doi:10.3389/fcimb.2021.614963)
Supplement: Supplementary file 6 [file Table_5.doc]

**Supplementary Table 5.** Primers and target genes used in this study. (Copy right obtained from Shen et al., 2015)

| **Target gene(s)** | **Primer** | **Nucleotide sequence (5′-3′)** | **Annealing temperature (°C)** | **Reference** |
| --- | --- | --- | --- | --- |
| *stx1a*,*stx1c*,*stx1d* | Stx1com-F | (C/T)AGTTGAGGGGGGTAAAATG | 52 | [(Beutin et al., 2007)](https://www.sciencedirect.com/science/article/pii/S0740002015000313#bib3) |
|  | Stx1com-R | CG(A/G)AAAATAAC(C/T)TCGCTGAATC |  |  |
| *stx2a*,*stx2b*, *stx2c*, *stx2dact*, *stx2e*,*stx2g* | LP43 | ATCCTATTCCCGGGAGTTTACG | 57 | [(Beutin et al., 2007)](https://www.sciencedirect.com/science/article/pii/S0740002015000313#bib3) |
|  | LP44 | GCGTCATCGTATACACAGGAGC |  |  |
| *stx2a*, *stx2c*, *stx2dact*, *stx2g* | GK3 | ATGAAGAAGATGTTTATG | 52 | [(Beutin et al., 2007)](https://www.sciencedirect.com/science/article/pii/S0740002015000313#bib3) |
|  | GK4 | TCAGTCATTATTAAACTG |  |  |
| *stx2dact* | Stx2dact | CTTTATATACAACGGGTG | 54 | [(Zheng et al., 2008)](https://www.sciencedirect.com/science/article/pii/S0740002015000313#bib60) |
|  | CKS1 | CTGAATTGTGACACAGATTAC |  |  |
| *stx2e* | stx2e-F | ATGAAGAAGATGTTTATAGCG | 62 | [(Beutin et al., 2007)](https://www.sciencedirect.com/science/article/pii/S0740002015000313#bib3) |
|  | stx2e-R | GTTAAACTTCACCTGGGCAAAG |  |  |
| *stx2b* | stx2b-F | AGGGCCCACTCTTTAAATACATCC | 52 | [(Beutin et al., 2007)](https://www.sciencedirect.com/science/article/pii/S0740002015000313#bib3) |
|  | stx2b-R | CGTCATTCCTGTTAACTGTGCG |  |  |
| *stx2f* | stx2f-F | AGATTGGGCGTCATTCACTGGTTG | 57 | [(Beutin et al., 2007)](https://www.sciencedirect.com/science/article/pii/S0740002015000313#bib3) |
|  | stx2f-R | TACTTTAATGGCCGCCCTGTCTCC |  |  |
| *eae* | eae229-F | CTGAACCAGATCGTAACGGC | 58 | [(Xia et al., 2010)](https://www.sciencedirect.com/science/article/pii/S0740002015000313#bib58) |
|  | eae229-R | TGATAAGCTGCAGTCGAATCC |  |  |
| *e-hlyA* | hlyA-F | AGCCGGAACAGTTCTCTCAG | 58 | [(Xia et al., 2010)](https://www.sciencedirect.com/science/article/pii/S0740002015000313#bib58) |
|  | hlyA-R | CCAGCATAACAGCCGATGT |  |  |
| *eae* | eae-F | AGGATATTCTTTCTCTGAATA | 57 | [(Tramuta et al., 2008)](https://www.sciencedirect.com/science/article/pii/S0740002015000313#bib56) |
|  | eae-R | ATATYTATTTGCWGSVCCCCAT |  |  |
| *eae*γ1 | gamma1-F | AAAACCGCGGAGATGACTTC | 60 | [(Mora et al., 2007)](https://www.sciencedirect.com/science/article/pii/S0740002015000313#bib40) |
|  | gamma1-R | AGAACGCTGCTCACTAGATGTC |  |  |
| *eae*ε1 | epsilon1-F | AAAACCGCGGAGATGACTTC | 66 | [(Mora et al., 2007)](https://www.sciencedirect.com/science/article/pii/S0740002015000313#bib40) |
|  | epsilon1-R | AGCTCACTCGTAGATGACGGCAAGCG |  |  |
