# Supplementary material for: Role of Recent Therapeutic Applications and the Infection Strategies of Shiga Toxin-Producing Escherichia coli
Source: Front Cell Infect Microbiol. 2021 Jun 29;11:614963. doi: 10.3389/fcimb.2021.614963 (PMC8276698; doi:10.3389/fcimb.2021.614963)
Supplement: Supplementary file 7 [file Table_6.doc]

**Supplementary Table 6.** Summary of antimicrobial resistance in STEC, as described in previous reports. (Copy right obtained from Bianca Amézquita-López et al., 2018).

| **Antimicrobial class** | **Domestic animal host** | **Function inhibited** | **Antimicrobial agent** | **Reference** |
| --- | --- | --- | --- | --- |
| Aminoglycosides | Cattle, Sheep | Bacterial protein synthesis | Amikacin | Amézquita-López et al 2016; Schroeder et al 2002 |
|  | Sheep |  | Gentamicin |  |
|  | Cattle, Sheep |  | Kanamycin |  |
| β-Lactamase inhibitors | Cattle | Cell wall synthesis; some β-lactamases | Amoxicillin – Clavulanic acid | Iweriebor et al 2015 |
| Cephems (parenteral) | Cattle, Sheep | Cell wall synthesis | Cephalothin |  |
|  | Cattle |  | Cefoperazone |  |
|  | Cattle |  | Ceftazidime |  |
|  | Cattle |  | Ceftriaxone |  |
| Folate pathway inhibitors | Cattle, Chicken, Pig, Turkey | Folic acid synthesis | Trimethoprim-Sulfamethoxazole | Bai et al 2016; Srinivasan et al 2007;  Uemura et al 2003 |
| Fosfomycins | Cattle, Pig | Enzymes involved in cell wall synthesis | Fosfomycin |  |
| Lipopeptides | Cattle, Chicken, Pig, Turkey | Bacterial membrane permeability | Colistin |  |
| Macrolides | Pig | Bacterial protein synthesis | Erythromycin |  |
| Penems | Sheep | Cell wall synthesis | Imipenem | Khan et al 2002; C.M.  Schroeder et al 2002 |
| Penicillins | Cattle, Chicken, Sheep | Cell wall synthesis | Ampicillin |  |
| Phenicols | Cattle, Sheep | RNA synthesis | Chloramphenicol |  |
| Quinolones | Cattle, Turkey | DNA synthesis | Ciprofloxacin |  |
|  | Cattle, Chicken, Pig, Turkey |  | Nalidixic acid |  |
| Tetracyclines | Pig, Sheep | Bacterial protein synthesis | Tetracycline | Bai et al 2016; Uemura et al 2003 |
