# Supplementary material for: Role of Recent Therapeutic Applications and the Infection Strategies of Shiga Toxin-Producing Escherichia coli
Source: Front Cell Infect Microbiol. 2021 Jun 29;11:614963. doi: 10.3389/fcimb.2021.614963 (PMC8276698; doi:10.3389/fcimb.2021.614963)
Supplement: Supplementary file 8 [file Table_7.doc]

**Supplementary Table 7.** Characteristics and/or associated functions of virulence genes. (Copy right obtained from Bianca Amézquita-López et al., 2018)

| **Target gene** | **Location** | **Characteristics and/or associated functions** |
| --- | --- | --- |
| **a) Hemolysins** | | |
| *ehxA* | pO157 plasmid | Enterohemolysin; produces small turbid zones of lysed red blood cells |
| *hlyA* | Chromosome | α-hemolysin; produces large clear zones of lysed red blood cells |
| *sheA* | Chromosome | induced hemolysin; found in pathogenic and non-pathogenic *E. coli* |
| **b) Adhesins** | | |
| *eae* | LEE region | Intimin; forms attaching and effacing lesions |
| *saa* | pO113 plasmid | STEC autoagglutinating adhesion; associated with non-O157 LEE-negative |
| **c) Effectors** | | |
| *ent/espL2* | O-Island 122 | Effector; alters cytoskeleton in human cells |
| *espK* | Prophage Sp6 | Effector; unknown function |
| *espN* | Prophage Sp6 | Effector; unknown function |
| *espP* | pO157 plasmid | Extracellular serine protease |
| *katP* | pO157 plasmid | EHEC catalase-peroxidase |
| *nleA* | O-Island 71 | Effector; disrupts protein secretion |
| *nleB* | O-Island 122 | Effector; interfere with inflammatory signaling pathways |
| *nleE* | O-Island 122 | Effector; interfere with inflammatory signaling pathways |
| *nleH1-2* | O-Island 71 | Effector; interfere with inflammatory signaling pathways |
| **4) Cytotoxins** | | |
| *stx*1a | Chromosome | *stx*1 prototype; 1000 times less cytotoxic than *stx*2a, repressed by iron |
| *stx*1c | Chromosome | *stx* variant linked to mild symptoms in humans; common in ovine STEC |
| *stx*1d | Chromosome | *stx* variant not associated with a particular food source |
| *stx*2a | Chromosome | *stx*2 prototype; linked to severe HUS in humans |
| *stx*2b | Chromosome | *stx* variant linked to *eae*-negative STEC and mild disease in humans |
| *stx*2c | Chromosome | *stx* variant linked to diarrhea and HUS in humans |
| *stx*2d | Chromosome | *stx* variant found in highly virulent strains; *stx* activity increased by elastase |
| *stx*2e | Chromosome | *stx* variant responsible for edema in pigs; rare in human disease |
| *stx*2f | Chromosome | *stx* variant isolated from pigeon; rare in human disease |
| *stx*2g | Chromosome | *stx* variant common in bovine STEC |
| *subA* | pO113 plasmid | Subtilase cytotoxin; triggers apoptosis in human cells |
| **5) O-antigens** | | |
| *wzy*O26 | Chromosome | *E. coli* O26 O-antigen polymerase |
| *wzy*O45 | Chromosome | *E. coli* O45 O-antigen polymerase |
| *wzx*O91 | Chromosome | *E. coli* O91 O-antigen flippase |
| *wzy*O103 | Chromosome | *E. coli* O103 O-antigen polymerase |
| *wzy*O104 | Chromosome | *E. coli* O104 O-antigen polymerase |
| *wzy*O111 | Chromosome | *E. coli* O111 O-antigen polymerase |
| *wzy*O113 | Chromosome | *E. coli* O113 O-antigen polymerase |
| *wzy*O121 | Chromosome | *E. coli* O121 O-antigen polymerase |
| *wzy*O128 | Chromosome | *E. coli* O128 O-antigen polymerase |
| *wzy*O145 | Chromosome | *E. coli* O145 O-antigen polymerase |
| *wzy*O157 | Chromosome | *E. coli* O157 O-antigen polymerase |
| **6) H-antigens** | | |
| *fliC*H2 | Chromosome | *E. coli* flagellar H2 antigen |
| *fliC*H7 | Chromosome | *E. coli* flagellar H7 antigen |
| *fliC*H8 | Chromosome | *E. coli* flagellar H8 antigen |
| *fliC*H11 | Chromosome | *E. coli* flagellar H11 antigen |
| *fliC*H19 | Chromosome | *E. coli* flagellar H19 antigen |
| *fliC*H21 | Chromosome | *E. coli* flagellar H21 antigen |
