# Supplementary material for: Role of Recent Therapeutic Applications and the Infection Strategies of Shiga Toxin-Producing Escherichia coli
Source: Front Cell Infect Microbiol. 2021 Jun 29;11:614963. doi: 10.3389/fcimb.2021.614963 (PMC8276698; doi:10.3389/fcimb.2021.614963)
Supplement: Supplementary file 9 [file Table_8.doc]

**Supplementary Table 8.** Probiotics and their sources. (Copyright obtained from Kerry et al., 2018)

| **Strain** | **Commercial products** | **Source** |
| --- | --- | --- |
| *Lactobacillus acidophilus NCFM*  *Bifidobacterium lactis HN019 (DR10)*  *Lactobacillus rhamnosus HN001 (DR20)* | Sold as ingredient | Danisco (Madison, WI) |
| *Saccharomyces cerevisiae boulardii* | Florastor | Biocodex (Creswell, OR) |
| *Bifidobacterium infantis 35,264* | Align | Procter and Gamble (Mason, OH) |
| *Lactobacillus fermentum VRI003 (PCC)* | Sold as ingredient | Probiomics (Eveleigh, Australia) |
| *Lactobacillus rhamnosus R0011 Lactobacillus acidophilus R0052* | Sold as ingredient | Institut Rosell (Montreal, Canada) |
| *Lactobacillus acidophilus LA5 Lactobacillus paracasei CRL 431* | Sold as ingredient | Chr. Hansen (Milwaukee, WI) |
| *Bifidobacterium lactis Bb-12* | Sold as ingredient | Chr. Hansen (Milwaukee, WI) |
| *Lactobacillus casei strain Shirota Bifidobacterium breve strain Yakult* | Yakult | Yakult (Tokyo, Japan) |
| *Lactobacillus casei DN-114 001 (“L. caseiImmunitas”)* | DanActive fermented milk | Danone (Paris, France) |
| *Bifidobacterium animalis DN173 010 (“Bifidis regularis”)* | Activia yogurt | Dannon (Tarrytown, NY) |
| *Lactobacillus reuteri RC-14 Lactobacillus rhamnosus GR-1* | Femdophilus | Chr. Hansens (Milwaukee, WI) |
|  |  | Urex Biotech (London, Ontario, Canada) |
|  |  | Jarrow Formulas (Los Angeles, CA) |
| *Lactobacillus johnsonii Lj-1 (same as NCC533 and formerly Lactobacillus acidophilus La-1)* | LC1 | Nestlé (Lausanne, Switzerland) |
| *Lactobacillus plantarum 299 V* | Sold as ingredient; Good Belly juice product | Probi AB (Lund, Sweden); NextFoods (Boulder, Colorado) |
| *Lactobacillus rhamnosus 271* | Sold as ingredient | Probi AB (Lund, Sweden) |
| *Lactobacillus reuteri ATCC 55,730 (“L. reuteri Protectis”)* | BioGaia Probiotic chewable tablets or drops | Biogaia (Stockholm, Sweden) |
| *Lactobacillus rhamnosus GG (“LGG”)* | Culturelle; Dannon Danimals | Valio Dairy (Helsinki, Finland) |
|  |  | The Dannon Company (Tarrytown, NY) |
| *Lactobacillus rhamnosus LB21* | Sold as ingredient | Essum AB (Umeå, Sweden) |
| *Lactococcus lactis L1A* |  |  |
| *Lactobacillus salivarius UCC118* | – | University College Cork (Cork, Ireland) |
| *Bifidobacterium longum BB536* | Sold as ingredient | Morinaga Milk Industry Co. Ltd. (Zama-City, Japan) |
| *Lactobacillus acidophilus LB* | Sold as ingredient | Lacteol Laboratory (Houdan, France) |
| *Lactobacillus paracasei F19* | Sold as ingredient | Medipharm (Des Moines, Iowa) |
| *Lactobacillus paracasei 33* | Sold as ingredient | GenMont Biotech (Taiwan) |
| *Lactobacillus rhamnosus GM-020* |  |  |
| *Lactobacillus paracasei GMNL-33* |  |  |
| *Lactobacillus plantarum OM* | Sold as ingredient | Bio-Energy Systems, Inc. (Kalispell, MT) |
| *Bacillus coagulans BC30* | Sustenex, Digestive Advantage and sold as ingredient | Ganeden Biotech Inc. (Cleveland, Ohio) |
| *Streptococcus oralis KJ3 Streptococcus uberis KJ2* | ProBiora3 | Oragenics Inc. (Alachua, FL) |
|  | EvoraPlus |  |
| *Lactobacilli rhamnosus PBO1 Lactobacilli gasseri EB01* | EcoVag | [Bifodan (Denmark), www.ecovag.com](http://www.ecovag.com/) |
